# Impact of race-neutral global reference equations on spirometry interpretation in healthy children in The Gambia

**DOI:** 10.5588/ijtldopen.24.0224

**Published:** 2024-09-01

**Authors:** E. Nkereuwem, V.F. Edem, O. Owolabi, M. Genekah, S.A. Owusu, E.D. McCollum, B. Kampmann, T. Togun

**Affiliations:** ^1^ Vaccines and Immunity Theme, MRC Unit The Gambia at the London School of Hygiene & Tropical Medicine, Fajara, The Gambia;; ^2^ Faculty of Infectious and Tropical Diseases, London School of Hygiene & Tropical Medicine, London, UK;; ^3^ Global Program in Pediatric Respiratory Sciences, Eudowood Division of Pediatric Respiratory Sciences, Department of Pediatrics, Johns Hopkins School of Medicine, Baltimore, MD, USA;; ^4^ Charité Centre for Global Health, Institute of International Health, Berlin, Germany;; ^5^ TB Centre, London School of Hygiene & Tropical Medicine, London, UK.

**Keywords:** lung function, reference equation, child, adolescent, Global Lung Function Initiative, GLI_2022_

Dear Editor,

Accurate interpretation of spirometry results is crucial for diagnosing and managing respiratory diseases, particularly in children. This interpretation relies on reference equations that predict lung function based on factors like age, sex, height, and, historically, race.^1^ In 2012, the Global Lung Function Initiative (GLI) introduced race-specific reference equations (GLI_2012_), aiming for standardised spirometry interpretation across populations.^2^ However, these equations were primarily developed using data from healthy individuals in North America, Europe and Asia and lacked representation from sub-Saharan Africa. This raises concerns about the accuracy of these equations in interpreting lung function for all populations. The inclusion of race in spirometry equations is a topic of debate due to its potential for bias. Using race-adjusted equations in underrepresented populations can lead to incorrect diagnosis and treatment of lung diseases.^3^ Relying on race-adjusted equations could also worsen already existing racial disparities in healthcare access and outcomes.^4^ To address this, the GLI released new race-neutral GLI_2022_ equations, which rely only on age, sex, and height.^5^ However, the impact of these revised race-neutral equations on lung function interpretation in children and adolescents from sub-Saharan Africa is yet to be described. Our objective was to evaluate the impact of the GLI_2022_ race-neutral equations on the interpretation of lung function in healthy Gambian children compared to the race-specific GLI_2012_ equations. Through this evaluation, we aimed to contribute to a more comprehensive understanding of their accuracy and their potential impact on clinical practice.

We performed a secondary analysis of spirometry data from 91 children and adolescents who participated as a healthy comparison group in a comparative study that assessed the prevalence of residual respiratory impairment in children after completion of TB treatment.^6^ The participants were aged 5–18 years and resided in the Western Region of The Gambia. They had no recent respiratory tract infections, no history of smoking and no known chronic respiratory conditions. Spirometry was conducted in accordance with the ERS/ATS (European Respiratory Society/American Thoracic Society) guidelines using a calibrated portable Easy on-PC spirometer (ndd, Zurich, Switzerland).^2^ We derived *z*-scores for forced expiratory volume in one second (zFEV_1_), forced vital capacity (zFVC), and the ratio of FEV_1_ to forced vital capacity (FVC) using the GLI_2012_ and GLI_2022_ reference equations.^2,5^ We considered the equation a good fit if the average *z*-score and standard deviation were not significantly different from zero and one, respectively. We defined the spirometry pattern using the 2022 ERS/ATS guidelines.^7^ This study was approved by the Gambian Government and MRC joint ethics committee, reference number 17747. Participants gave informed consent to participate in the study before taking part.

Of the 91 healthy children and adolescents enrolled, five (5.5%) did not meet the quality criteria for spirometry and were excluded. The median age of participants was 11.9 years (interquartile range 8.1‒13.7); 34 (39.5%) were female, and 28 (32.6%) reported exposure to environmental tobacco smoke (Supplementary Table S1). The GLI_2012_ ‘African American’ (mean ± SD zFEV_1_: –0.91 ± 0.87; zFVC: –0.97 ± 0.93), ‘Others/Mixed’ (zFEV_1_: –1.58 ± 0.87; zFVC: –1.71 ± 1.00), and ‘South-East Asian’ (zFEV_1_: –1.33 ± 0.88; zFVC: –1.23 ± 0.97) equations had a better fit for this group than the race-neutral GLI_2022_ equations (zFEV_1_: –1.62 ± 0.75; zFVC: –1.66 ± 0.79). Conversely, the GLI_2012_ ‘Caucasian’ and ‘North-East Asian’ equations performed worst, with zFEV_1_ and zFVC less than the GLI_2022_ estimates. However, the zFEV_1_/FVC ratio was similar across most reference equations, except for the GLI_2012_ ‘South-East Asian’ equation, which showed a significantly lower mean ratio (Figure 1 and Supplementary Table S2). The proportion of participants with abnormal spirometry results varied significantly across the reference equations. The GLI_2012_ ‘African American’ equations had the lowest proportion (27%), whereas the GLI_2012_ ‘North-East Asian’ equations had the highest proportion (83%). Additionally, the GLI_2022_ equation classified 26% and 19% more as abnormal spirometry than the ‘African American’ and ‘South-East Asian’ GLI_2012_ equations, respectively (Figure 2).

**Figure 1.**
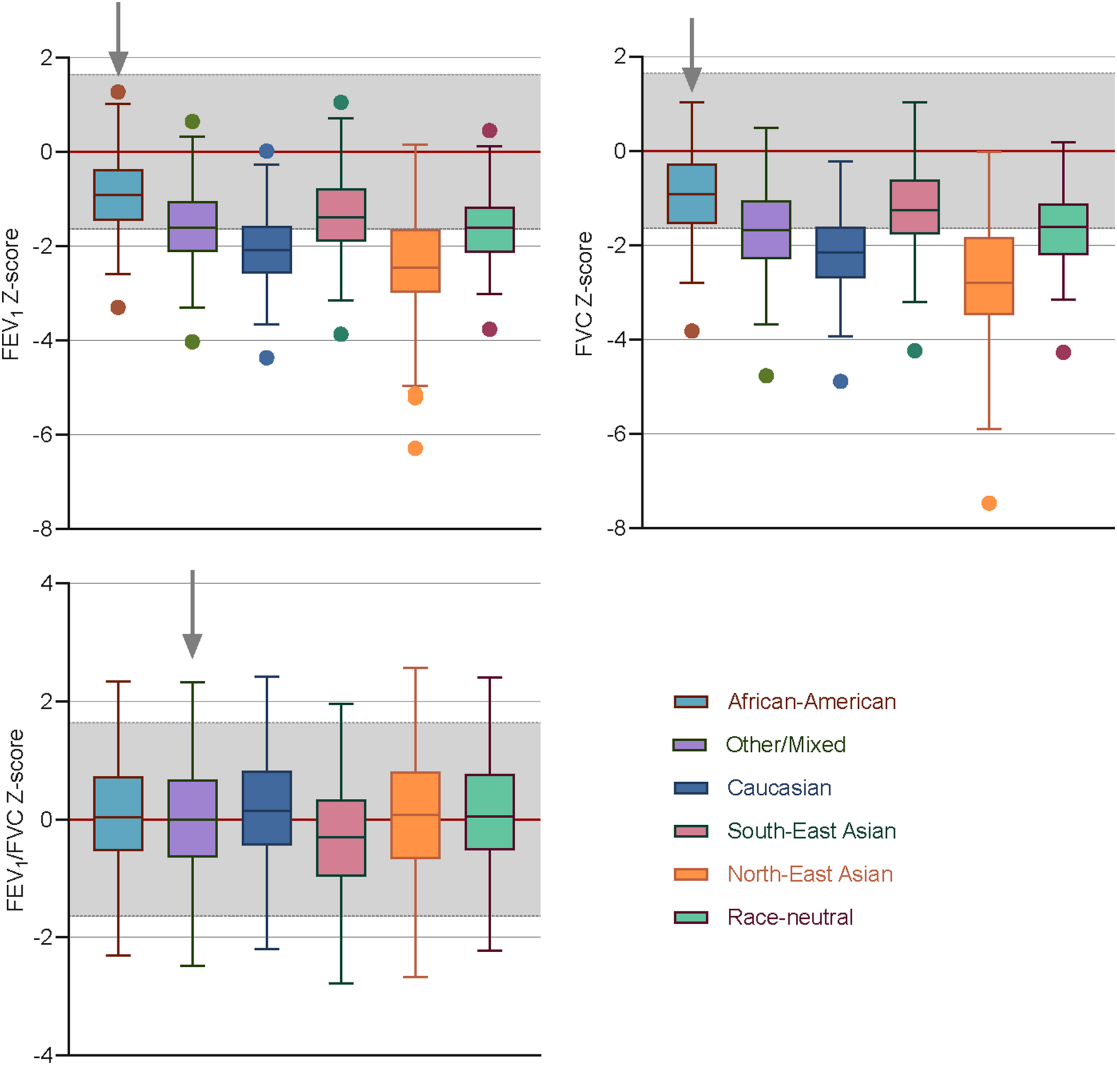
Distribution of *z*-scores of FEV_1_, FVC, and FEV_1_/FVC using the GLI_2012_ (African-American, Other/Mixed, Caucasian, South East Asian and North East Asian) reference equations and the GLI_2022_ race-neutral reference equation for the study population. The equations that resulted in the closest fit to a mean *z*-score of zero and a standard deviation of one were selected as the best fit (arrow). The shaded area is from –1.64 to +1.64 *z*-scores and represents the expected normal range for spirometry volumes. FEV_1_ = forced expiratory volume in one second; FVC = forced vital capacity; GLI_2012_ = 2012 Global Lung Function Initiative.

**Figure 2.**
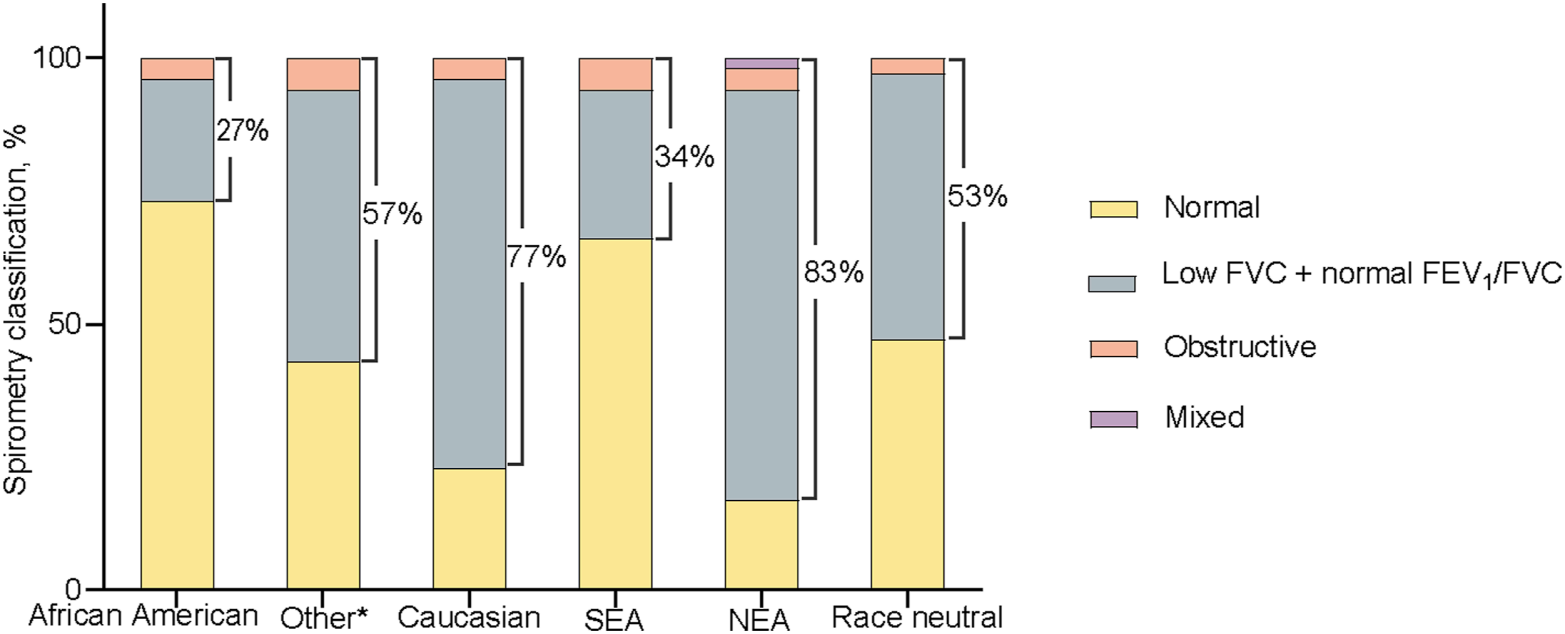
Stacked bar charts showing the spirometry outcome classification using the GLI_2012_ (African American, Other/Mixed, Caucasian, SEA and NEA) reference equations and the GLI_2022_ race-neutral reference equation for the study population. The proportion (%) of participants classified as abnormal using each reference equation is shown. *Or mixed race. SEA = South East Asian; NEA = North East Asian; GLI_2012_ = 2012 Global Lung Function Initiative.

Our study provides valuable insights into the challenges of applying race-neutral spirometry reference equations in Gambian children and adolescents. We observed that several GLI_2012_ equations had a better fit for this group than the race-neutral GLI_2022_ equations. Although using GLI_2012_ ‘African American’ and ‘South-East Asian’ equations resulted in fewer participants with abnormal spirometry, all reference equations classified a significant proportion of these healthy participants as abnormal. These findings align with a recent systematic review which demonstrated that healthy West African populations showed a poor fit to all GLI_2012_ reference equations.^8^ Furthermore, this raises important concerns regarding the suitability of the race-neutral equations for this population and highlights the need for further research into region-specific adjustments or the development of entirely new reference equations. It is worth noting that similar challenges have also been observed in other populations.^5,9^ For example, there have been ongoing debates about using race-based reference equations in the United States, particularly for African American and Latino populations.^4^ Studies have shown that race-based reference equations, such as those developed for African Americans, can result in differences in spirometry interpretation and misclassification of lung function impairment.^10^ Some researchers suggest that using race-based equations may contribute to health disparities by perpetuating the notion of inherent physiological differences between races.^11^ However, others argue that race-based reference equations are necessary to account for genetic and physiologic differences between populations.^12^ It is a complex issue that requires further investigation and consideration of social, political, and ethical implications. Beyond genetics, various factors such as environmental exposures, nutritional deficiencies, anthropometry and socio-economic disparities likely contribute to differences in lung function.^13^ For example, a study of healthy, multi-ethnic children in London, UK, found that race-specific GLI_2012_ equations described lung function in these children as normal.^14^ This suggests that ethnicity may be an important factor influencing spirometry when considering local contexts and environmental factors in the interpretation of what ‘normal’ spirometry is.

We acknowledge that this is a proof-of-concept study. Additionally, spirometry alone cannot infer the presence of restrictive lung function abnormalities.^7^ However, relying solely on spirometry reference equations to define ‘normal’ lung function overlooks the intricate interplay of genetic and environmental factors that influence respiratory health.^15^ A holistic and patient-centred approach to managing respiratory illnesses should incorporate the evaluation of spirometry trends over time alongside a thorough clinical assessment. Importantly, spirometry reference equations should not be used as stand-alone tools for diagnosing or labelling individuals.

In conclusion, although some GLI_2012_ equations appeared to be a better fit for our sample group than the GLI_2022_ equations, our findings suggest that all existing GLI equations have the potential to misclassify spirometry results in healthy Gambian children and adolescents. This highlights the need for more nuanced and context-specific approaches to assess lung function. This should take into account not only race but also environmental and social factors that can influence lung health. By doing this, we can gain a better understanding of respiratory health disparities and develop effective strategies to address them.

## Supplementary Material


