# Supplementary material for: Impact of race-neutral global reference equations on spirometry interpretation in healthy children in The Gambia
Source: IJTLD Open. 2024 Sep 1;1(9):418–21. doi: 10.5588/ijtldopen.24.0224 (PMC11409167; doi:10.5588/ijtldopen.24.0224)
Supplement: Supplementary file 1 [file ijtldopen24-0224_supplementarydata1.pdf]

Supplementary Material

Impact of race-neutral GLI global reference equations on spirometry interpretation in healthy Gambian children

Esin Nkereuwem[1,2], Victory Fabian Edem[1], Olumuyiwa Owolabi[1], Monica Genekah[1], Sheila Ageiwaa Owusu[1,2], Eric D. McCollum[3], Beate Kampmann[1,2,4], Toyin Togun[1,2,5]

[1] Vaccines and Immunity Theme, MRC Unit The Gambia at the London School of Hygiene and Tropical Medicine.

[2] Faculty of Infectious and Tropical Diseases, London School of Hygiene and Tropical Medicine, London, UK

[3] Global Program in Pediatric Respiratory Sciences, Eudowood Division of Pediatric Respiratory Sciences, Department of Pediatrics, Johns Hopkins School of Medicine, Baltimore, United States

[4] Charité Centre for Global Health, Institute of International Health, Berlin, Germany

[5] TB Centre, London School of Hygiene and Tropical Medicine, London, UK

Running head: GLI Global Equations in Gambian Children

Supplementary Table 1. Participant characteristics

**Variable**	**Participants enrolled (n=86)**
Age, median (IQR)	11.9 (8.1, 13.7)
Female sex	34 (39.5)
Environmental tobacco smoke	28 (32.6)
Type of cooking fuel	
Wood	58 (67.4)
Charcoal	26 (30.2)
Gas	2 (2.3)
BMI-for-age *z*-score, median (IQR)	-1.23 (-1.81, -0.46)
Height-for-age *z*-score	-0.17 (-1.01, 0.28)
Underweight	18 (20.9)
Stunted	6 (6.9)
Data are presented as n (%) unless otherwise indicated.	

Supplementary Table 2. Mean z-scores (SD) for each GLI reference equation (N=86)

**Reference range**	**FEV_1 z-score (mean ± SD)**	**FVC z-score (mean ± SD)**	**FEV_1/FVC (mean ± SD)**
GLI_{2012}			
African American	-0.91 ± 0.87	-0.97 ± 0.93	0.08 ± 0.92
Others/mixed	-1.58 ± 0.87	-1.71 ± 1.00	0.02 ± 0.96
Caucasian	-2.05 ± 0.82	-2.18 ± 0.89	0.18 ± 0.92
South-East Asian	-1.33 ± 0.88	-1.23 ± 0.97	-0.29 ± 0.96
North-East Asian	-2.43 ± 1.21	-2.80 ± 1.39	0.10 ± 1.08
GLI_{2022} race-neutral	-1.62 ± 0.75	-1.66 ± 0.79	0.12 ± 0.92

FEV_1: forced expiratory volume in one second; FVC: forced vital capacity; SD: standard deviation.
